# Conventional and conservative management of placenta accreta is two ends of a single continuum: A report of three cases and literature review

**DOI:** 10.1002/ccr3.1717

**Published:** 2018-07-13

**Authors:** Yousaf Latif Khan, Arooba Rahim, Javed Gardezi, Mariam Iqbal, Zahira Hassan, Sumbal Altaf, Shahzad Bhatti

**Affiliations:** ^1^ Department of Gynecology and Obstetrics Hameed Latif Hospital 14 – Abu Bakar Block New Garden Town Lahore Pakistan; ^2^ Department of Gynecology and Obstetrics Rashid Latif Medical College 35‐KM Ferozepur Road Lahore Pakistan; ^3^ Department of Cellular Pathology Royal Free Hospital London UK; ^4^ Department of Medical Education Rashid Latif Medical College 35‐KM Ferozepur Road Lahore Pakistan; ^5^ Department of Human Genetics and Molecular Biology University of Health Sciences Lahore Pakistan; ^6^ Lahore Institute of Fertility and Endocrinology Hameed Latif Hospital Lahore 14 – Abu Bakar Block New Garden Town Lahore Pakistan

**Keywords:** Abnormal placentation, cesarean hysterectomy, cesarean section, conservative management, placenta accreta

## Abstract

Placenta accreta (PA) is a critical condition that represents a significant source of morbidity and mortality observed in women with multiple prior cesarean sections. Precise prenatal identification of affected pregnancies permits optimal obstetric management. Antenatal diagnosis leads to less blood loss and a requirement for blood transfusion than women diagnose during cesarean section.

## 1 INTRODUCTION

Placenta accreta (PA) is a serious obstetric disorder that is characterized by deep penetration of the villi which are abnormally attached to the myometrium of the uterus. This obstructs its complete separation during the third stage of labor which induces continued bleeding, and have potentially life‐threatening for the mother.^1^ Most commonly, it is a consequence of a partial or complete absence of the compact and spongy layer known as the decidua basalis, and misdevelopment of the fibrinoid Nitabuch's layer which lies between the boundary zone of the thick endometrium and the cytotrophoblastic shell in the placenta.^2^ The placental villi attached and are only slightly embedded into the myometrium in Placenta accreta (more than 70% cases), deeply invade into the myometrium in placenta increta (13%) and invade the perimetrium even infiltrating adjacent structures of the pelvic floor in placenta percreta (5%).^3^

Clinically, severe complications of PA include severe obstetric hemorrhage leading to disseminated intravascular coagulopathy (DIC), iatrogenic injury to the ureters, bladder, bowel, respiratory distress syndrome (RDS), acute transfusion reactions, electrolyte imbalance, and renal failure. In women with PA, the expected blood loss at delivery is 3000‐5000 mL, and maternal deaths rate is as high as 7%. About 90% of patients require a blood transfusion with 40% requiring more than ten units of packed cell transfusion.^4^ Different methods have been employed to manage the PA, ranging from uterine conservation, which involves leaving the placenta in situ, to conventional hysterectomy. Classical cesarean sections (C‐sections) prevent the excessive bleeding by leaving the adherent placenta in situ and by adopting strategic planning with a comprehensive analysis that aids the reduction in maternal morbidity and mortality rates.^5^ We report three consecutive cases of PA, treated in our hospital between 10 January 2016, and 14 July 2016, for analysis and discussion. Personal interviews were conducted to get information regarding the clinical and demographic parameters along with the cesarean history of patients. There were no maternal deaths, and all patients delivered healthy babies.

### 1.1 Case 1

**Figure 1:**
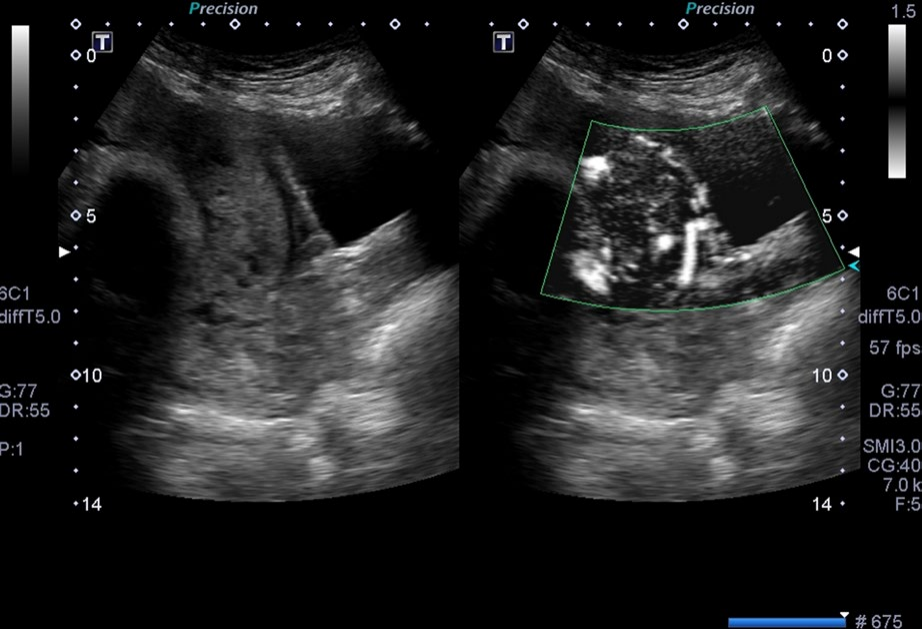
Sagittal transvaginal ultrasound Image shows abnormal irregular bladder uterine serosa boarder

A 32‐year‐old lady, gravida 5, para 4 (G5P4) was accepted as a referred case in the Department of Obstetrics and Gynecology due to abnormal placentation diagnosed during a routine ultrasound late in pregnancy at 29 weeks of gestation. She had a 3‐year medical history of previous cesarean delivery due to placenta previa. We followed a high index of precision for the clinical diagnosis of invasive placentation. A trans‐abdominal ultrasound examination using a 6‐1.9 MHz trans‐abdominal probe (Toshiba Artida PVT‐375BT, Inc, Yokohama, Japan) revealed a viable pregnancy with normal amniotic fluid and appropriate fetal sonographic biometric parameters. We assessed the grade and number of placenta lacunae in accordance with Feinberg's criteria. Transabdominal ultrasonography revealed the presence of intraplacental lacunae along with loss of retroplacental clear zone and irregular disruption of bladder–uterine serosa interface with least myometrial thickness (<1 mm) Figure 1. The bladder was moderately filled to evaluate its involvement better. The patient was informed about all treatment options, and their possible consequences including hysterectomy as uterine preservation is a fertility‐sparing option but feasible only in selected cases. Following this, an elective cesarean section was scheduled at 36 weeks of gestation. On the scheduled due date of the delivery, the patient was taken to the operating theatre after preoperative preparation and blood arrangements. After exploratory laparotomy, intra‐abdominal findings included an array of invasive placentation within the lower uterine segment. After initial displacement of the bladder had been achieved, a transverse uterine incision was proceeded above the lower section of the uterus by avoiding the placental bed. A healthy baby girl of 5 pounds, 8 ounces was delivered. Subsequently, there was a severe hemorrhage, and a cesarean hysterectomy was decided upon, to be performed immediately. The placenta only penetrated up to the myometrium and reached the serosa to some extent, along with the formation of several hematomas on the bladder mucosa while dissecting which required stitching. The total estimated blood loss was 6 L. Intraoperatively, we transfused six units of whole blood and 1500 mL free‐frozen plasma (FFPs). The postoperative recovery was smooth, and Foley's catheter was retained for 5 days. The patient was discharged on the sixth postoperative day in favorable recovery conditions.

### 1.2 Case 2

**Figure 2:**
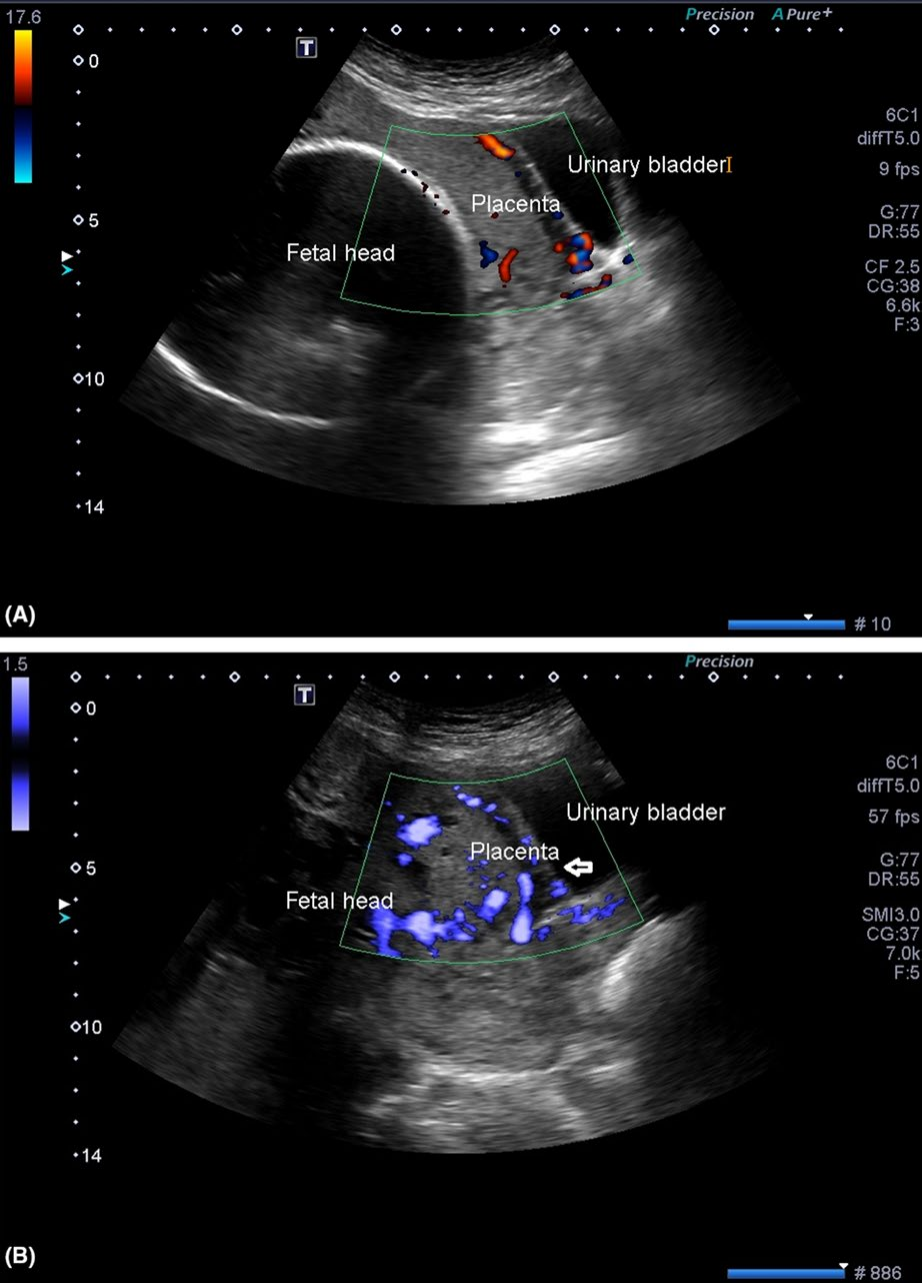
Sagittal transvaginal ultrasound image shows (A) Thinning of the myometrium in the lower uterine segment with few prominent vessels along the scar line. (B) The placenta is seen extending to the serosal surface of the bladder

**Figure 3:**
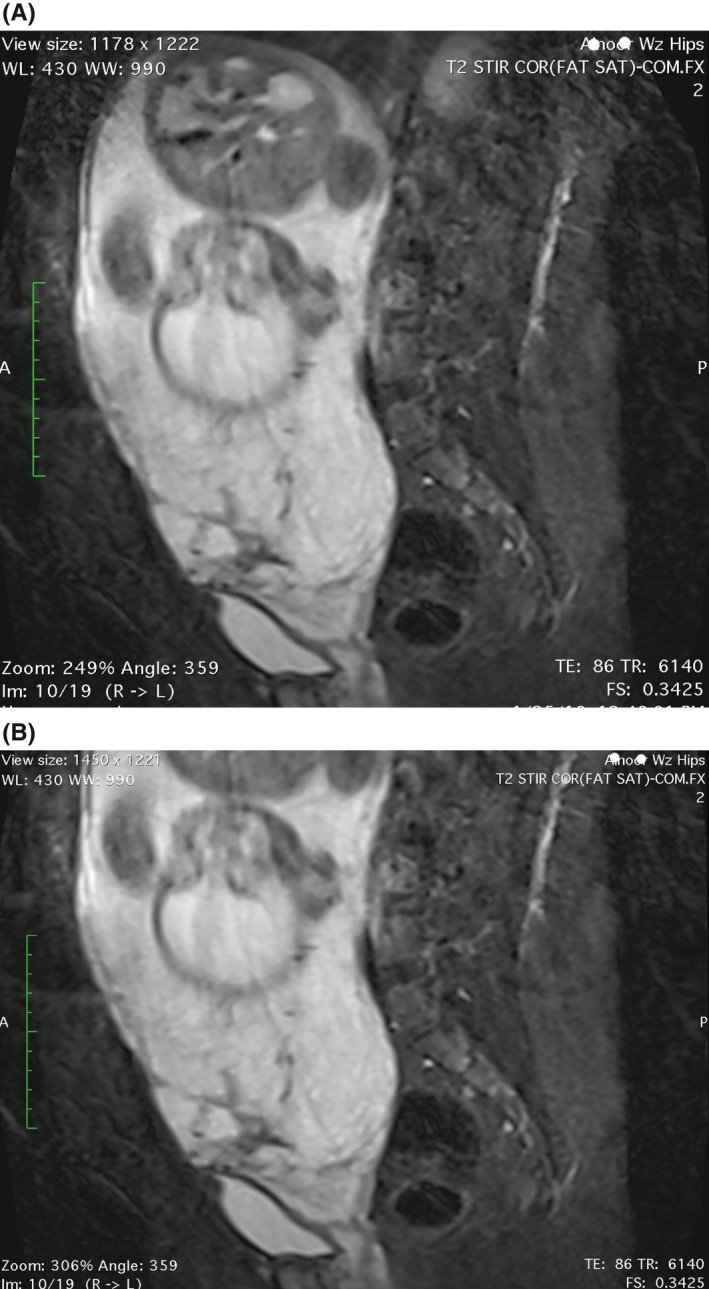
Magnetic resonance imaging (MRI) Pelvis without contrast: (A) sagittal T2‐weighted MR image shows bulging of the uterus and tenting of the bladder. (B) Placenta lies anteriorly along with lower uterine segment completely covering the internal os

A healthy 35‐year‐old woman with a history of 2 elective cesarean sections and one miscarriage (G4 P2 A1) was accepted as a referred case to our hospital. The patient was admitted for the elective cesarean section at 37 weeks of gestation. She was referred from the rural primary care hospital where routine ultrasonography revealed low‐lying placentation. A Doppler ultrasound at 35 weeks showed complete covering of the cervical os which was attached to the scar with incessant hemorrhagic lesions in the inner anterior myometrium, indicative signs of PA (Figure 2). She had diagnosed placentation in the lower uterine segment and had given a history of irregular, painless bleeding of the vagina during the fifth and seventh months of gestation. Further, magnetic resonance imaging (MRI) was performed, images still revealed that the placenta was completely covering the cervical os and suspected implantation of placental villi penetrating the full thickness of the myometrium, which further extending posteriorly up to the bladder wall and anteriorly displayed vascular engorgement (Figure 3). On the day of surgery, an expert urologic surgeon had been involved along with the interventional cardiology team. During the cesarean, the uterus was opened along the midline in upper segment of uterus, and a baby of the 3 kg was delivered with a good Apgar score. Due to invasive placentation and bleeding, cesarean hysterectomy was proceeded immediately after the birth of the baby. The lower uterine segment was noted to be relatively thin but was covered by a plexus of deep vessels which spread over the bladder near the broad ligament which was covered by peritoneum. The bladder was opened and repaired due to continuous bleeding as the placenta extended to the bladder. Left‐sided salpingo‐oophorectomy was also performed due to constant bleeding from the left tubo‐ovarian ligament. The estimated blood loss was approximately 3500 mL during the procedure. Afterward, three units of whole blood were transfused, and two hemaccel infusions were given. The patient was kept on inotropic support in postoperative ICU for 24 hours. Later, the patient was moved to the surgical ward and discharged from the hospital on the 5th postoperative day. Foley's catheter was retained for 2 weeks, and a smooth postpartum recovery was observed.

### 1.3 Case 3

**Figure 4:**
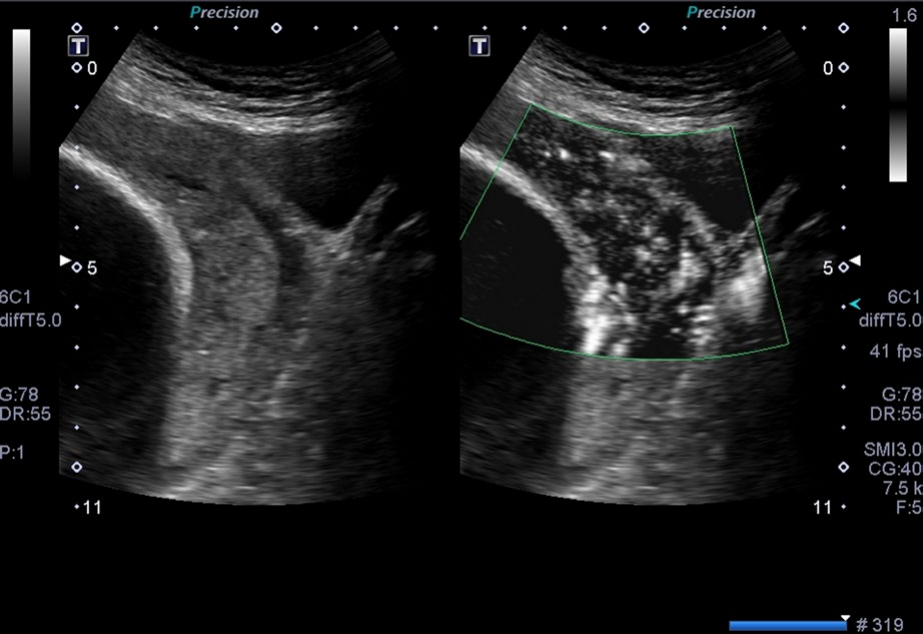
On Sagittal transvaginal ultrasound image placenta is seen extending to the serosal surface of the bladder

A 38‐year‐old patient, gravida 4, para 2 (G4P2 +1) with two cesarean sections and a history of tubal pregnancy was admitted for the elective cesarean section at 36 + 2 weeks, ultrasound from an outside private hospital revealed full placenta previa and suspected placenta accreta (Figure 4). PA was further diagnosed on MRI. The patient had strongly wished for future fertility, and we informed him and her husband about the expected risks associated with this conservative treatment. After full preparation in theatre, the uterus was opened in the upper segment, sparing the placenta. A baby of 2.4 kg with a good Apgar score was delivered. A portion of the placenta was delivered immediately after the birth of the baby. However, there was excessive bleeding in the lower segment with a part of placenta adhered to the uterine wall. Several stitches were taken in lower uterine segment, and the B‐Lynch suture was applied after which bleeding stopped. The estimated blood loss was 2 L. Two units of whole blood and 1‐unit of FFP were transfused during surgery while 1‐unit whole blood was given postoperatively. A single dose of MTX (Methotrexate) injection (25 mg/mL) was given commencing from the first day after the cesarean sections (C‐sections) along with broad spectrum antibiotic cover. Postoperative recovery was satisfactory, and the patient was discharged on the 4th postnatal day. On the 30th postoperative day, the patient was re‐admitted due to vaginal bleeding, an immediate D & C (dilation and curettage) was performed, and the residual placental mass was completely removed. After 1 week, ultrasonography confirmed an empty uterine cavity and no further complications were noted.

## 2 DISCUSSION

There has been a high incidence of PA during the past several decades due to the extensively high number of C‐sections which is one of the commonest risk factors for PA, given its association with myometrial tissue damage caused by previous cesarean delivery.^6^ Other potential risk factors which contributed significantly to the development of PA are advanced maternal age, multiparity, or any condition resulting in myometrial tissue damage, followed by secondary collagen repair (such as any prior myomectomy), vigorously forced curettage, submucosal pedunculated leiomyomas, endometrial ablation, and uterine fibroid embolization.^7^ Because of tissue damage, decidua basalis replaced by loose connective tissue characterized by degenerative lesions, fibrosis, inadequate of blood supply, and local hypoxia which further commenced deep infiltration of trophoblast. This infiltration of trophoblast is particularly noticeable when it covers the entire area of the cesarean cicatrix. When a cell has inadequate oxygen supply, it produces a hypoxia‐induced factor (HIF, ie, transcriptional factor) to stimulate the release of endothelial growth factor‐A (VEGF‐A) which in turn binds to VEGF‐A receptors on endothelial cells, activating a tyrosine kinase signaling pathway responsible for vasculogenesis. However, if hypoxia persists, it causes incorrect expression and instability of the secretion of VEGF, PGF (placental growth factor), and sFlt‐1 (soluble fms‐like tyrosine kinase ‐1), respectively. That leads to excessive secretion of VEGF, which appears to play a vital role in the pathology and the invasiveness of extravillous trophoblast cells in the mother placental space.^8^

The occurrence of PA has augmented over the past century from 1/7000 to 1/2500 deliveries due to extensive cesarean deliveries. Notably, every previous cesarean birth increases the risk of both, placenta previa and PA, respectively.^8^ Conventionally, the option of treatment for aggressive placentation has been associated with hysterectomy because of the potentially high risk of postpartum hemorrhage. It is a possible causal link between all the developed surgical complications including injuries abutting critical structures, massive blood transfusions, DIC, and substantial death rates.^9^ Over the last decade, the process of management has altered from existing conventional approach to avoid leaving any portion of the placenta in utero, to a more conservative concept of leaving the placenta in situ. Some widely cited studies have confirmed that the conservative approach is linked with fewer blood transfusions and hysterectomies.^10^ In our cases, the women, driven by several issues, signify a strong desire to attempt to care for the uterus, even though they were well informed about the diagnosis, risks to their health, and even life. However, after the diagnosis of PA, it is not possible to save the uterus, which is extensively challenging due to higher degrees of hemorrhage, development of disseminated intravascular coagulopathies (DIC), and succeeding surgical operations such as hysterectomies carried out under more complex hemodynamic situations in intensive care. Maternal deaths from PA are approximately 6%‐7% irrespective of the type of surgical procedures performed. The timing of the cases necessitating surgical procedure was not unexpected. Classic C‐sections were scheduled and carried out in an appropriate mode between 36 and 37 weeks of gestation. An interdisciplinary team, including interventional cardiologists and urologists, has been involved in preventing bleeding as well as other medical complications during the surgical procedure.

In diagnosed cases of PA and high‐risk patients of different age groups, preoperative preparation has markedly improved the outcome, including allowing for tertiary care centers for handling high‐risk cases. The most senior obstetricians, the surgical expertise of the vascular and trauma surgeons, senior anesthetist, and blood bank outline the most critical part of the team dealing with such cases. Before starting a surgery, required blood products including red blood products, FFPs, platelets, and cryoprecipitates should be available.^11^ Cell salvage techniques are increasingly being used globally but not practiced in our country. After injectable corticosteroid medication, mothers deliver late‐preterm babies in overall good health conditions, without complications in the development of the newborn. Starting surgery for planned peripartum hysterectomy is strongly supported by midline vertical incision. Once entering the abdomen, inspection for the placental invasion and its extent is the first step. The most typical site for placenta percreta is through the anterior uterine wall entering the bladder. Other sites may include a lateral extension of the placenta reaching the ureters. The uterus is opened at the point while avoiding the placenta. For this, prior placental localization by imaging techniques is critical. After delivery, minimal handling of the placenta and proceeding to hysterectomy decreases morbidity. Use of the uterotonics at this point is controversial as these may cause placental disruption, but at the same time preventing uterine atony.^12^

Subsequently, after delivery, another critical aspect is embolization of internal iliac arteries, which is effective significantly for the control of intraoperative blood losses, without intrusion into the placenta. However, separation of placenta was associated with profuse hemorrhage even after ligation and clamping of internal iliac arteries resulting in an expected blood loss of 2000‐5000 mL and mean time for the procedure were 3 hours and 10 minutes.^13^ The reason for this is because the uterine blood supply is also characterized by another plexus of blood vessels running down from the abdominal aorta, such as external iliac arteries, and femoral arteries. After ligation at the levels of internal iliac arties, these arteries can maintain a steady flow of blood in the area under surgical resection. Furthermore, incorrect placentation is responsible for stimulation of VEGF receptors which are considered to be one of the most critical events that lead to the deep vasculogenesis around the urinary bladder which in turn may stimulate the uncontrolled bleeding.^14^

Conventionally, hysterectomy is the most commonly performed procedure during cesarean section. It has been a critical element in the treatment of the PA and always a matter of choice to reduce the obstetric blood loss in the patients reported with substantial invasions and those who do not need to preserve fertility in the future. As a result, recent studies compared the earlier cases supplemented with new approaches when reporting incidence and consequences of cesarean hysterectomy.

The primary endpoint in the treatment of PA is uterine conservation for future fertility. In most cases, it appears that the conservative management of PA is successful in avoiding hysterectomies, but there is always a potential risk that could be expected in morbidity management. If a conservative approach is applied, close monitoring during the follow‐up is mandatory for detecting any complications which may emerge weeks to months after delivery or C‐section. In this context, our hospital provides 24‐hour emergency facilities, including ultrasound Doppler, proper blood bank, and availability of leading obstetricians to deal with any complications. Kelekciet et al^15^ and his coworkers involved in the prospective multicenter study demonstrated a combined surgical procedure in the conservative treatment of PA of twelve patients. A choice of conservative and nonconservative treatment had been given after obtaining hysterectomy consent from patients. The patients, who had chosen conservative treatment, retained the placenta in situ as it is the standard procedure. Out of all, just a single patient preferred a nonconservative method. The 11 patients who underwent conventional procedures did not need a hysterectomy and preservation of the uterus had been achieved in all patients without any abundant vaginal bleeding. Gupta et al^16^ reported 29 patients diagnosed antenatally by Doppler ultrasound. The previous history showed one cesarean was reported in 16 (55.1%) and two or more cesareans were found in 7 (24.1%) cases. The placenta was partially separated in 34.4% of cases and retained in 37.9% of cases, while 27.5% of women underwent emergency obstetric hysterectomies. Uterine embolization was performed in 37.9% of cases and managed conservatively by leaving the placenta in situ. Beta‐human chorionic gonadotrophin (beta‐hCG) levels were monitored along with Doppler ultrasound in a 5‐month follow‐up period. Conclusively, internal iliac arteries embolization, multidisciplinary team involvement, and leaving placenta in situ were the best conservative management in such cases. Chaudhari et al^17^ reported in a retrospective cum prospective study that in second gravida patients of age 26‐28 years have a high ratio (1.32% per 1000 pregnancies) of morbidity adherent placenta. About 90% of patients in this study had a previous history of C‐section. Forty percent have elective deliveries between 35 and 38 weeks of gestation, while in 27% uterus was preserved and prophylactic balloon replacement in the internal lilac artery followed by classical C‐section coupled with a postoperative dose of MTX (Methotrexate). The estimated total blood loss was about 1000‐2000 mL. However, patients kept on passing small fragments of degenerated placenta for the first few weeks, and regular menstruation continued after a few months. The above results are also supported by Farasatinasab et al^18^ in their retrospective review demonstrated the usage MTX as an adjuvant antimetabolite drug for the conservative management of PA to avoid any major surgery and for natural conception in the future. Another prospective study conducted by Lin et al^19^ demonstrated 24 stable patients with PA were observed efficacy following MTX management therapy. Approximately in 33.3% patients, the expulsion of the residual placenta takes place spontaneously while dilation and curettage (D & C) in 45.8% of the patient. The use of MTX therapy results in a gradual decrease in the beta‐hCG, and complete resorption of the retained placenta occurred totally within 5.7 months.

Alternatively, Framarino‐Dei‐Malatesta et al^10^ reported two cases of conservative management of PA which were managed successfully by leaving the placenta in situ and a low dose administration of MTX injection postoperatively to avoid significant adverse effects such as internal systemic complications, hepatic toxicity, kidney failure, and myeloablation. On the fifth postoperative day after MTX administration, ultrasonography revealed a progressive reduction in placental surface and complete placental expulsion commenced after 3‐4 weeks. Above studies support the optimum conservative management approach of PA that appears to be safe and effective in comparison with extirpative managements which lead to greater blood transfusion and hysterectomy rates.

So far effective management of the attached residual placenta remains indistinct as the widely used treatment is performing a hysterectomy due to the plexus of deep vessels distributed in the lower uterine segment. A significant drawback of conservative treatment of PA appears to be leaving the placenta in situ which may result in a loss of fertility, urinary tract infections, gastroenteritis, and bleeding, all of which present severe repercussions.

## 3 CONCLUSION

Over the past decade, with the increase in cesarean delivery, the incidence of abnormal placentation and severe blood loss as a result of abnormal vascularization is the leading cause of maternal morbidity and mortality. Continuous improvements are markedly required to provide evidence‐based strategies for treating these women. Furthermore, developments in medical procedures are required for diagnosis and the improved clinical implication of recent advances in order to increase our understanding of PA. Additionally, all hospitals should develop methods for women in regard to risk management and strategic treatment of PA to avail the opportunity to decrease morbidity and mortality. Nevertheless, poorly implemented procedures result in higher liability for a hospital than not having specific systems at all.

## ETHICAL APPROVAL AND CONSENT TO PARTICIPATE

The study was approved by the Institutional Ethical Committee (IEC) in accordance with Helsinki Declarations.

## CONSENT FOR PUBLICATION

Written informed consent was obtained from the patients for publication of this case report.

## CONFLICT OF INTEREST

None declared.

## AUTHORSHIP

YLK and JG: were involved in surgery as main operators. AR: involved in the surgery and in the postoperative management. MI and SA: were involved in postoperative management. SB: was involved in case of analysis, writing the manuscript, literature review, and critically reviewed this article. ZH: interpreted the patient image of MRI and reviewed this article. All authors read and approved the final manuscript.
